# **Vertebrate scavenging patterns during extreme winter conditions in North Dakota**

**DOI:** 10.1038/s41598-025-28834-5

**Published:** 2025-12-29

**Authors:** Lavinia Iancu, Nicolette Ras

**Affiliations:** Forensic Science Program, University of North Dakota, 221 Centennial Drive, Grand Forks, ND 58202 USA

**Keywords:** Decomposition, Winter, Extreme environment, Vertebrate scavenging, North dakota, Ecology, Ecology, Zoology

## Abstract

The decomposition process for above-the-ground human remains is usually divided into five stages: fresh, bloat, active decay, advanced decay, and skeletal remains. The chronology of these stages is influenced by numerous factors, including environmental temperature, microbial proliferation, necrophagous insect species activity, and vertebrate scavenging. The current research aimed to investigate the vertebrate scavenger’s activity during North Dakota winters, using pig carcasses as human analogues. The carcasses were placed outdoors in December 2022, on Mekinock Field Station, Grand Forks County ND, and monitored until the snow melted in May 2023. The monitoring took place daily via trail cameras with motion sensors, while the temperature was recorded hourly using temperature data loggers. The temperature fluctuated between − 32℃ and 30.3℃ along the six experimental months, with a snow cover of up to 130 cm. While the carcasses were covered by snow most of the time, the main vertebrate scavengers observed, represented by coyote (*Canis latrans* Say, 1823) and the red fox (*Vulpes vulpes* (Linnaeus, 1758)), dug up corridors to reach and consume the tissues, mostly after sunset. Field based monitoring studies are of tremendous help to understand the factors that induce variation in the chronology of the decomposition stages, invertebrate and vertebrate diversity, and colonization patterns. The current data will be of use to the forensic science field, as it relates to death investigations and search and recovery efforts, by providing an inventory of the primary vertebrate scavengers in this far North location.

**Supplementary Information:**

The online version contains supplementary material available at 10.1038/s41598-025-28834-5.

## Introduction

The decomposition process begins in the gastrointestinal tract^1^, and, during warmer months, is primarily driven by microbial and insect activity. Vertebrate scavengers also play a significant role during the decomposition process, particularly during the winter months when insects are absent^2^. Although scavengers have been studied in ecological contexts since the 70’s^3,4^, most research focuses on food webs rather than forensic implications^5–7^. Moreover, predation and scavenging are discussed separately, though, most predators can scavenge in various circumstances, with more energy being transferred per scavenger link when compared to predation^5,8^. The decomposition process is influenced by multiple biotic and abiotic factors, and vertebrate scavenging will introduce additional variability by accelerating tissue removal and scattering of remains. These actions will alter the overall decomposition pattern, having significant forensic implications.

Competition for decomposed remains occurs not only among vertebrate scavengers but also between scavengers, microbes, and insects during warmer months. During higher temperatures, microbial proliferation can make the entire carrion undesirable to most scavengers^2^, while chemical cues are the main factor of carrion detection and attraction, acting as attractants or repellents depending on concentration^9–11^. While during the warmer months the interspecific competition between vertebrate scavengers, microbes, and insects is more intense, it decreases in intensity during the winter months, especially at sub-zero temperatures when insect activity ceases. Although these interspecific interactions are investigated and discussed in the ecology arena, analyzing them from a forensic perspective will provide a better understanding for interpreting outdoor death scenes involving scavenging.

To understand scavengers behavior, experimental studies have used different animal carcasses such as chickens or pigs, across various geographical locations^12,13^ and environments, including farmlands and undisturbed natural habitats^14^. As previously demonstrated, scavengers efficacy varies by season and habitat^3^, and one key factor influencing the frequency of scavenging is carrion availability. The environment and carrion accessibility further affect scavenging activity^2^. Often, scavenging is observed on animals that died from accidents, malnutrition, or diseases, rather than from a predator kill, with ungulates representing the main source of carrion in many terrestrial environments^5^. Irrespective of the cause of death, carrion constitutes an ephemeral food resource in a given environment and attracts a range of obligate and facultative scavengers^2,5,7^.

During winter periods with high snow depths, scavengers may increase their reliance on carrion as a food source^15^. Although most available carcasses are subject to scavenging, quantifying the amount of carrion consumed before decomposition remains challenging. Consequently, systematic monitoring using trail cameras can provide crucial information about the scavenging behavior, which is highly relevant for death investigations. Since a large proportion of terrestrial vertebrates can be facultative scavengers, the presence and scavenging debut and activity must be considered when investigating an outdoor death scene, as animal scavenging is a frequent occurrence in forensic cases.

Most of the studies focusing on scavenging from the forensic science perspective are reported from North America^16–22^, however, the topic is under-researched^23^. Most of the studies are from Texas, Colorado, Tennessee, and North Carolina, with no records for scavenging data for North Dakota winter months^24^. Scavenging of human remains affects the microbiome and insect activity, hindering forensic investigations by removing the soft tissues, disarticulating the skeletal remains and even remove personal effects^25,26^. These scavenging activities can produce changes that could lead to the obliteration of trauma sites and misinterpretation of events related to the death of the individual. For example, in a previous study from southern Texas, animal claw marks were misinterpreted as defense wounds^27^. Moreover, monitoring studies related to vertebrate scavenging are extremely valuable in gathering data on scavengers species, temperature, season, and environment^28,29^.

According to the latest data^30^, 2,300 Americans are reported missing every day, while 4,400 unidentified bodies are recovered every year from both urban and rural outdoor areas. Given the high number of cases that end in fatalities, it is important to have data on all factors that might influence the decomposition of human remains, including vertebrate scavengers. Furthermore, understanding scavenging in a forensic context will also provide valuable data for postmortem interval (PMI) estimation, as in several cases scavenging caused errors in the estimation of the time elapsed since death, by accelerating the decomposition process via removal of soft tissues^31,32^, or delaying the process by removing or consuming the insects^33^.

Most of the time, detection dogs are used to locate human remains^34^. Data on scavengers and scavenging behavior could enhance the search strategies used by human detection dogs’ teams, by improving and developing updated search and recovery protocols.

In light of these implications, and considering that vertebrate scavenging data can provide valuable information regarding the PMI estimation through predictable scavenging patterns^17^, the current research aimed to address two questions: (1) What are the primary scavengers of outdoor remains in Grand Forks County, ND? (2) When are these scavengers active during the extreme winter season?

## Results

### Environmental data

**Fig. 1:**
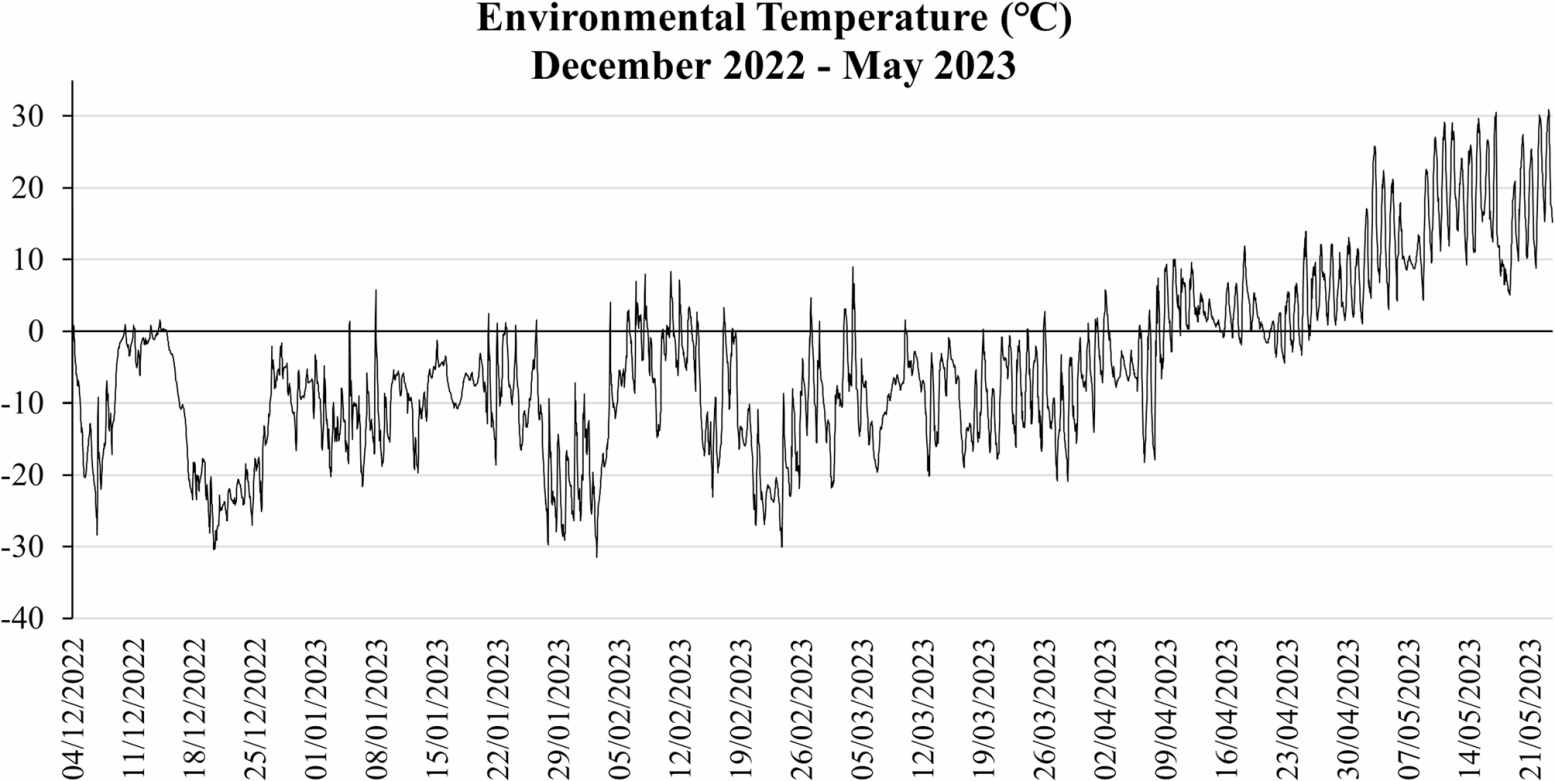
Environmental temperature variation from December 2022 to May 2023.

**Fig. 2:**
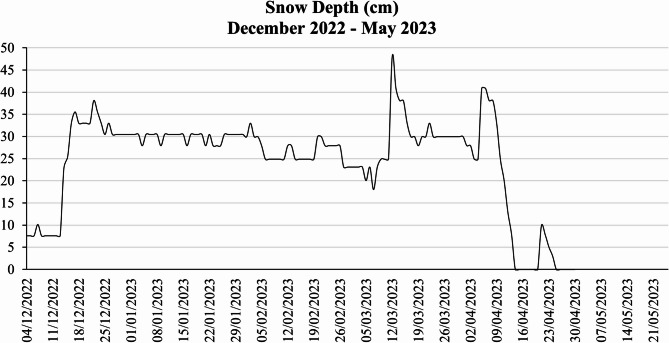
Snow precipitation records from December 2022 to May 2023.

During the investigated months the temperature recorded a minimum of -32℃ and a maximum of 30.3℃ (Fig. 1). During the first four experimental months, December 2022 – March 2023, the temperature records were sub-zero, with most values in the range of -10℃ and − 20℃. December and February were characterized by very low temperatures, dropping to -32℃ (Fig. 1), and significant snowfall (Fig. 2).

During early December the snow precipitation was relatively low (7–10 cm), after which a gradual increase can be observed, with a high snow precipitation recorded in mid-December (38.1 cm) (Fig. 2). After mid-December the snow precipitation was relatively constant, reaching a maximum record of 48 cm. The last snow was recorded between April 21 and 24, decreasing from 10 to 3 cm (Fig. 2). Total precipitation during the experimental time frame was higher than the historical average of the region^35^, with a snow cover of up to 130 cm in certain field areas.

## Vertebrate scavenging pattern

**Fig. 3:**
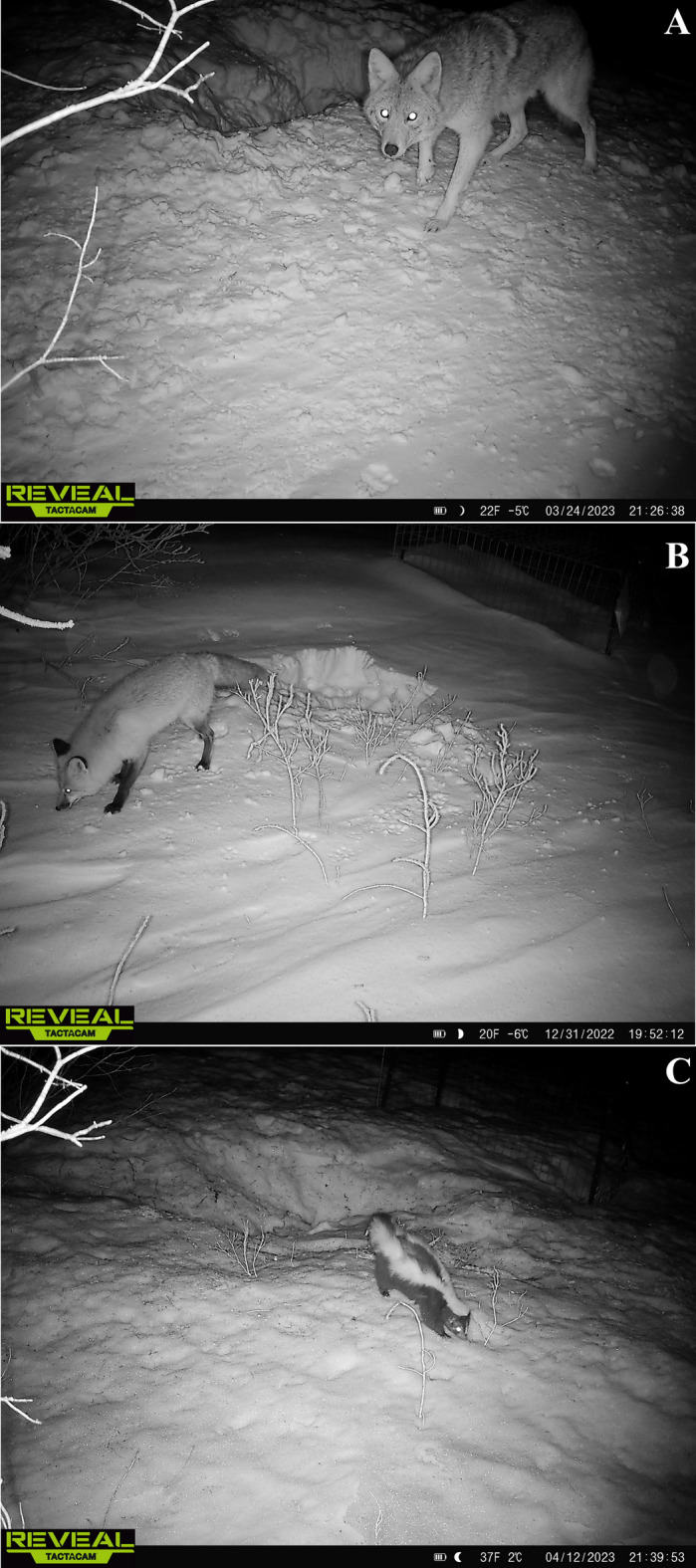
Main scavengers recorded during the experimental period: (**A**) *Canis latrans* Say, 1823; (**B**) *Vulpes vulpes* (Linnaeus, 1758); (**C**) *Mephitis mephitis* (Schreber, 1776).

The pig carcasses were placed in the field on December 5, 2022, at 13:50, and were covered by snow for most of the experimental time. Scavenging was observed starting on December 31, 2022, with the main scavengers being the coyote (*Canis latrans* Say, 1823), the red fox (*Vulpes vulpes* (Linnaeus, 1758)), and the skunk (*Mephitis mephitis* (Schreber, 1776)) (Fig. 3). Scavenging primarily occurred after sunset, with both canid scavengers often digging corridors to reach and consume the carcasses. The presence of these three species was expected, as they are commonly found in the Northern United States. A raccoon (*Procyon lotor* (Linnaeus, 1758)) and several blue jays (*Cyanocitta cristata* (Linnaeus, 1758)) were recorded as infrequent visitors (Supplementary video).

The removal of pig remains, including tissue and bones, was hindered by freezing conditions, resulting in all scavenging activities occurring at the initial carcass deployment area. Due to snow coverage, it was not always possible to note which side of the carcass was scavenged first. The winter conditions slowed decomposition, keeping the remains fresh and edible for a longer period.

**Fig. 4:**
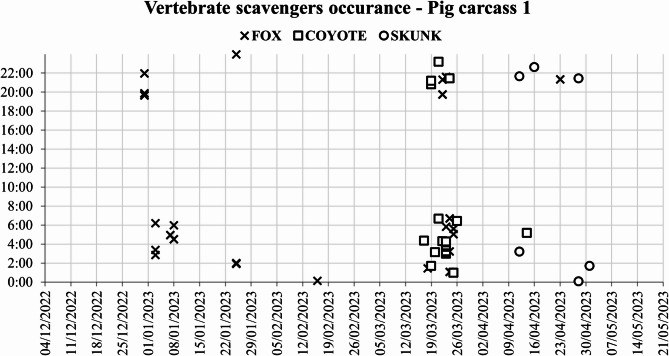
Occurrence of vertebrate scavengers at pig carcass 1 from December 2022 to May 2023.

From the 1800 photographs and 1200 videos analyzed, the red fox triggered the camera 50 times out of 150 for the first pig carcass, 315 times out of 768 for the second pig carcass, and 139 times out of 368 for the third pig carcass. These 504 camera triggers covered 13 distinct scavenging days for the first pig carcass (Fig. 4), 21 days for the second pig carcass, and 18 days for the third pig carcass, with scavenging hotspots clustered around the first and last week of January and the first week of February (Supplementary Figs. 1, 2 and 3). During this period, air temperatures ranged between − 20 and − 30 °C.

**Fig. 5:**
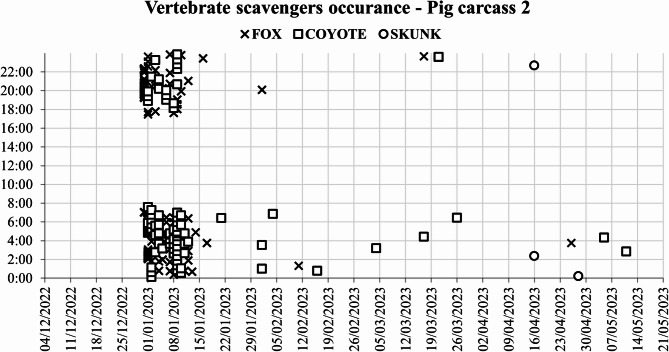
Occurrence of vertebrate scavengers at pig carcass 2 from December 2022 to May 2023.

The coyote triggered the camera 33 times for the first pig carcass, accounting for 9 distinct days. For the second pig carcass, 419 camera triggers accounted for 22 days (Fig. 5), and for the third pig carcass, 183 camera triggers corresponded to 18 distinct days. Coyote scavenging behavior was nocturnal, with a single late afternoon appearance.

**Fig. 6:**
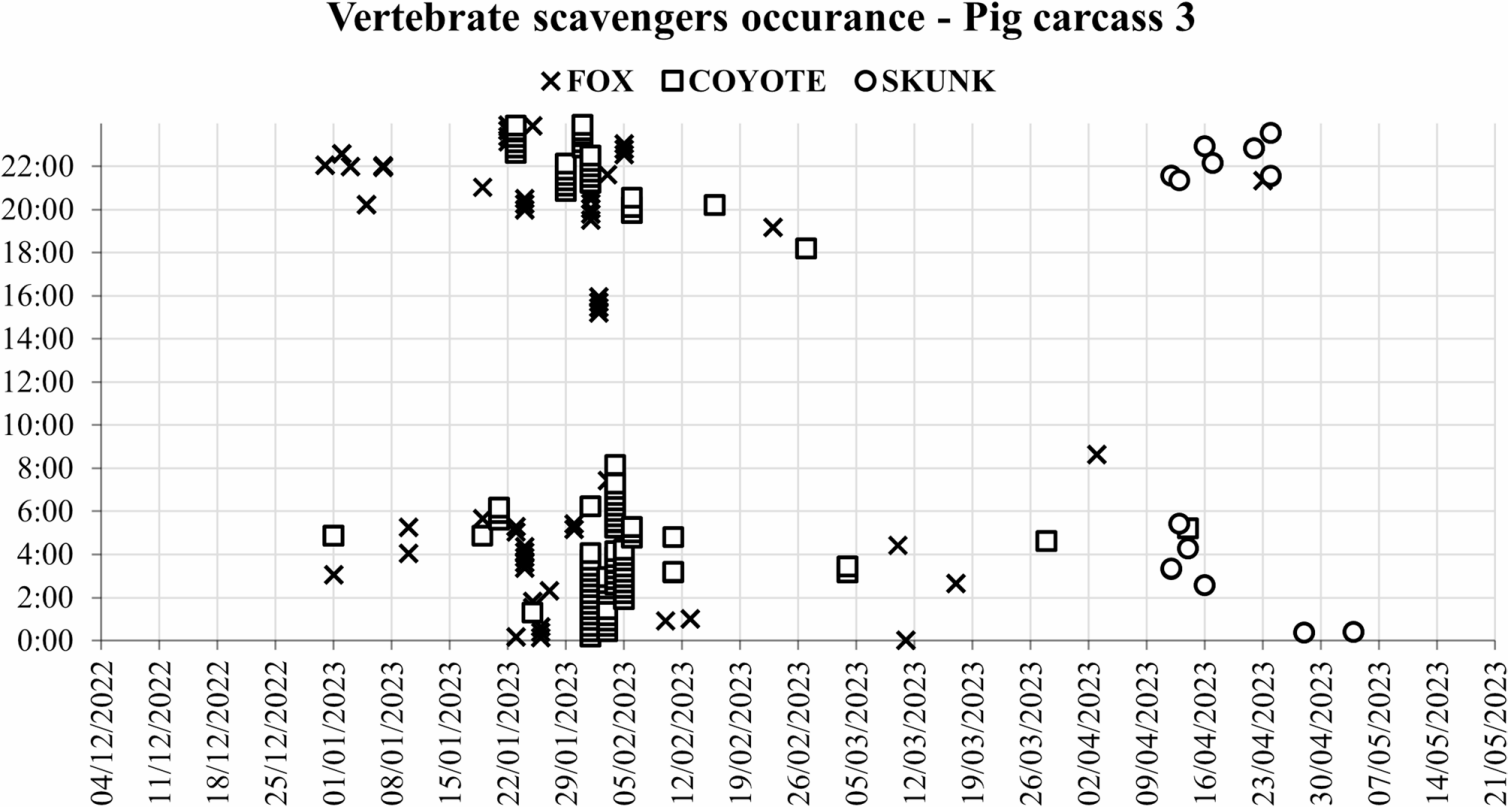
Occurrence of vertebrate scavengers at pig carcass 3 from December 2022 to May 2023.

Skunk activity towards the end of the scavenging period coincided with the end of winter (Fig. 6). This late presence is explained by the fact that skunks enter a torpor state from November until March, significantly reducing their activity to conserve energy and survive the harsh North Dakota winter conditions.

The scavengers primarily exhibited solitary behaviors, with the exception of coyotes, which scavenged in pairs during March and April. The most intense scavenging activity was recorded between 20:00 and 06:00. Scavenging occurred intermittently among different species. The red fox was the sole scavenger for the first pig carcass until late March (Fig. 6). In contrast, the second pig carcass was predominantly scavenged by coyotes. The third carcass experienced alternating scavenging between the red fox and coyotes, with distinct times recorded for each species. The chi-square test indicated a significant association between the pig carcasses and scavenger species (χ² = 56.67, df = 4, *p* < 0.001). Furthermore, the correspondence analysis confirmed the visual observations for the first pig carcass association with the red fox scavenging, followed by the second pig carcass balanced distribution with slight association with the coyote scavenging, and the third pig carcass with an intermediate distribution for both fox and coyote scavenging.

## Discussion

The current study aimed to provide key information related to vertebrate scavenging activities during North Dakota winter months, to be of use for death investigations in outdoor locations in Grand Forks County and other areas with similar environments.

The taphonomic impact of scavenging changes with season and habitat^36^. A few studies targeted the monitoring of scavengers’ activities in the wetlands^37^ and sunflower fields^38^ of North and South Dakota, primarily during mid and late summer. These early studies evaluated the scavenging activity on blackbirds, which are considered pests, causing significant crop damage in the Northern Great Plains. The research focused on the ecological impact of poisoned blackbirds, particularly on vertebrate scavengers. Both studies focused on the importance of understanding non-target animal mortalities that can be associated with agricultural pesticide applications. Further, the aim of one of these earlier studies was to assess the search efficiency and carcass removal by scavengers in cattail marshes^37^. It is noteworthy that the experiment took place from August to September 1987. The researchers analyzed the removal time data, defined as the time between carrion placement and complete removal^37^. The results suggested that the water depth influenced the removal times, however, the scavenger’s activity was substantial in the North Dakota’s cattail marshes, including the scavenger species involved. Further, in the second study^38^, the researchers focused on song bird carrion removal times from sunflower and corn fields in late summer and early spring. The rates of carcasses persistence varied between fields, emphasizing that the removal times are dependent on the season and habitat. This later study^38^ focused on areas from Grand Forks and Nelson counties (ND), and Miner and Brookings counties (SD). Coyotes, red foxes, and raccoons fed on the birds’ carcasses in spring and fall. Scavenging was more pronounced in the sunflower fields compared to harvested cornfields. The same scavenger species were recorded in the current study; however, the raccoon was not recorded as a dominant species, having a sporadic presence. Moreover, the skunk followed in the scavenging succession, after the fox and coyote, while the persistent snow cover and subzero temperatures significantly slowed decomposition.

As in the case of necrophagous insect species colonization, the vertebrate scavenging activity is influenced by biotic and abiotic factors^14^. When considering carrion removal in addition to inter- and intra-competition, and biotic and abiotic factors, the secondary scavengers’ densities within a specific habitat must be investigated and considered. In a recent study, the authors investigated the carrion removal rates by scavengers in a farmed region in Indiana, USA^14^. The study recorded raccoons and opossums as the main scavengers. The authors emphasized that the carrion competition is different in agricultural landscapes as compared to other habitats, and that the temperature influenced the carcass removal times, decreasing with an increase in temperature. This aspect is reflected by the current study, as the pig carcasses were not foraged immediately after field placement, but two weeks later, which corresponded with a drop in temperatures and an increase in snow accumulation. This could suggest that scavenging occurs when other food alternatives are no longer available. Additionally, habitat fragmentation must be investigated as well, as this will also affect scavenging patterns^14^. Noteworthy, the current research recorded only three main species responsible for the carcass forging, in agreement with similar studies showing that these species thrive in fragmented, human-altered landscapes^39–41^. In contrast to warmer environments, where carcass relocation or dispersal is common, the freezing conditions recorded during this experiment prevented the scavengers from removing large portions of tissues or bones, resulting in all feeding occurring at the original deposition site.

Obligate scavengers are very rare, when compared to facultative scavengers^2^. Vulture species are considered obligatory scavengers, as they are completely adapted to scavenge, and they can outcompete other vertebrate scavengers. In the current experiment, no vultures were recorded. Even though Turkey vultures can be found in North Dakota, including Grand Forks County, these species migrate south for the winter. It is important to consider that, if present, vultures can detect and consume carrion faster than other scavengers^5^. Moreover, vultures will not forage the carrion during nighttime, when other mammalian scavengers will be present^5^.

Several previous studies focused on carrion removal^14,37,38^ showed that this activity occurs in different percentages across different habitats. In the current experiment, no carrion removal was noted, which can be attributed to the carrion mass and frozen conditions. Smaller carrions are easier to remove from the field and carry away. During death investigations of bodies found in an open field, animal paths and dens should be searched for scattered remains, as well as personal belongings. As previously reported^42^, foxes can scatter remains 10 to 45 m from the deposition site.

In another earlier study in Northern Virgina USA^19^, experiments carried out on exposed and buried carcasses identified red foxes, opossums, skunks, raccoons, crows, and turkey vultures as scavengers. It is worth mentioning that scavengers avoided feeding on carcasses colonized by insects. Nevertheless, the scavenging behavior of foxes was nocturnal, as reported by the current results. A study performed in Ontario, Canada^43^ reported the coyote, red fox, fisher, pine marten, bald eagle, turkey vulture, and corvids as scavengers, and found that the season impacted the scavengers presence and the decomposition process, with a fast tissue consumption during the summer months.

Coyotes and foxes are more likely to scavenge exposed remains in a rural area like Grand Forks County. However, snow depth and temperature must be considered, as scavenging was predominantly observed during the lowest temperatures. As previously reported^42,44^, the coyote will focus on scavenging the remains where they are deposited, a behavior recorded in the current study as well. Moreover, both studies noted that the coyote presence has the potential to disrupt fox scavenging activities. Nevertheless, other previous study^43^ reported that the coyote was responsible for the dispersion of the remains, thus, these different behaviors must be considered when comparing data from different locations. The coyote scavenging behavior can expose soft tissues, otherwise inaccessible to the fox. The coyote was among the scavengers identified from a recent experiment in Yukon, Canada^45^, however, the study aimed to test the difference between carnivore and herbivore carcasses scavenging. The research revealed that the carnivore carcasses were scavenged later by the secondary scavengers to avoid parasites transmission and diseases.

Coyotes have been reported to interfere with the activity of other scavengers, like the bobcats^46^, and are considered a source of risk for other small scavengers. Coyotes will kill fox species and sometimes raccoons^46^. In the current experiment, the coyote presence might have reduced the feeding times of the red fox. For example, for the second pig carcass the fox activity declined from 310 camera triggers before coyote arrival to just 5 afterward. However, for the first and third pig carcasses, the fox activity remained stable after coyotes began scavenging. This can suggest that the influence of the coyote presence on fox scavenging behavior could be context dependent, influenced by carcass location or resource availability.

Skunk scavenging activities on human remains were previously reported from studies carried out in Colorado, USA, where they were observed feeding on tissues from the limbs^47^. While skunk scavenging is not often reported^47^, this species is very common in North America^48,49^, being less active during the winter months in the northern regions of the USA. In the current study, skunks were observed nocturnally, consistent to prior reports^47^. However, when they were observed near the remains, most of the soft tissue had already been consumed by coyotes and foxes.

The limitations of the current study are represented by the use of only one camera for each carcass and the usage of unclothed carcasses. Future studies on scavenging behavior should be region-specific to develop a local database for outdoor death scene investigations. Moreover, studies should consider the role of clothing, including the fiber type, as it can act as a barrier for scavengers, causing a reduction of the decomposition rate^50^.

## Materials and methods

### Experimental design

Three pig (*Sus domesticus* Erxleben, 1777) carcasses, each weighing 55 kg, were used as human analogues during the current winter field based scavenging study. The carcasses were placed in the field without protective cages and monitored from December 2022 until May 2023. This approach was based on a previous winter experiment (2021–2022)^51^, where carcasses were protected by cages, and scavengers’ presence was observed from March throughout the end of April. The current experiment aimed to observe the vertebrate scavengers’ activity without such barriers. The pig carcasses were euthanized by captive blitz bolt at a local pig farm and then transported to the experimental location within 45 min and placed in proximity to the cages used in the previous experiment, 20 m apart from one another and more than 30 m from the secondary road.

**Fig. 7:**
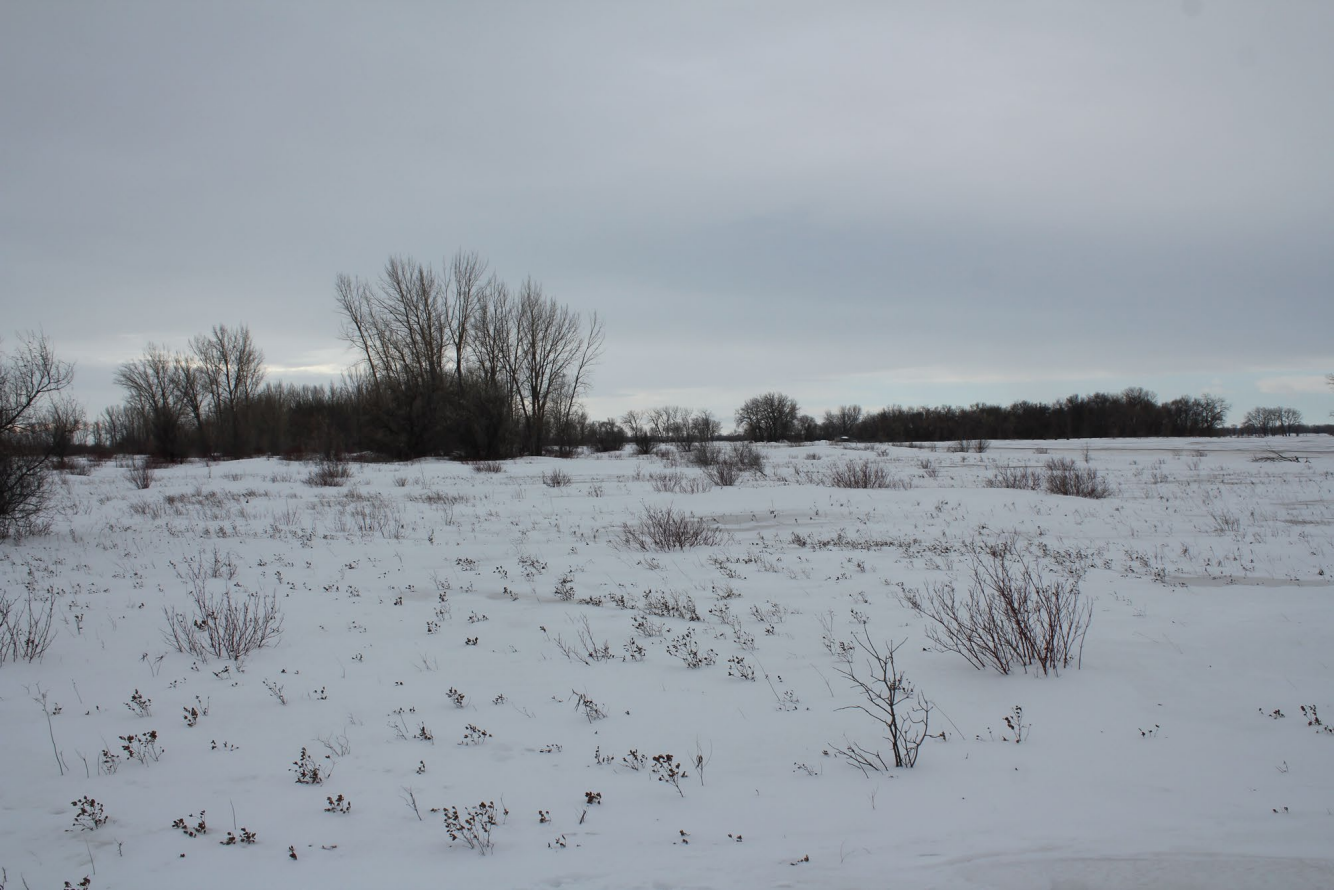
Mekinock Field Station, North Dakota, December 2022.

The study was conducted at Mekinock Field Station (47°57’11.5"N 97°25’42.3"W) (Fig. 7), Grand Forks County ND, with approval from the Field Station Committee. The Institutional Animal Care and Use Committee (IACUC) protocol approval was not required, as the pigs were euthanized at the pig farm prior to the study. Vertebrate scavenger activity was monitored passively via trail cameras, with no direct animal interaction. All methods were carried out in accordance with relevant institutional, national, and international guidelines and regulations.

### Field monitoring

For monitoring the decomposition and scavengers’ activity, trail cameras (TACTACAM Reveal) were placed at a height of 1.5 m, and 3 m away from each pig carcass, at a 30° angle. The trail cameras have a detection range of 29 m, with a trigger speed of less than ½ second, and minimal motion blur. Photographs and videos were triggered by motion sensors, with a Passive Infra-Red delay interval of 15 s, in addition to the preset ones taken at 12-hour intervals. Over the six-month period, 1800 photographs and 1200 videos were recorded and analyzed. The scavenging data, including dates and times, has been compiled into an Excel file. Cameras captured color photographs during the day and black and white photographs during the night.

Both photographs and videos were manually analyzed to record the presence times of scavengers. The data, including the specific days and hours when presence was detected, were documented and recorded in Microsoft Excel.

A chi-square test of independence was performed to assess the association between the pig carcasses and the scavenger species. Observed frequencies were compared to expected frequencies under the assumption of independence. Additionally, a correspondence analysis was conducted to visualize and further explore the relationship between the pig carcasses and the scavenger species. The analysis produced a two-dimensional solution summarizing the variation in the contingency table.

The hourly temperature was recorded with temperature data loggers (thermo button 22 L, Plug&Track, USA) placed 1.5 m above ground level, under each trail camera. Daily relative humidity and precipitation records were obtained from Grand Forks International Airport Station, located 10 km from the research site^35^. The winter conditions at Mekinock Field Station are typical for the northern Great Plains environment, with consistent extreme low temperatures and strong winds, which can frequently exceed 60 km/h^35^.

## Supplementary Information

Below is the link to the electronic supplementary material.

Supplementary Material 1

Supplementary Material 2

Supplementary Material 3

Supplementary Material 4

## Data Availability

All data generated or analyzed during this study are included in this published article.
