# Supplementary figures and images for: Vertebrate scavenging patterns during extreme winter conditions in North Dakota
Source: Sci Rep. 2025 Dec 29;15:44905. doi: 10.1038/s41598-025-28834-5 (PMC12749515; doi:10.1038/s41598-025-28834-5)

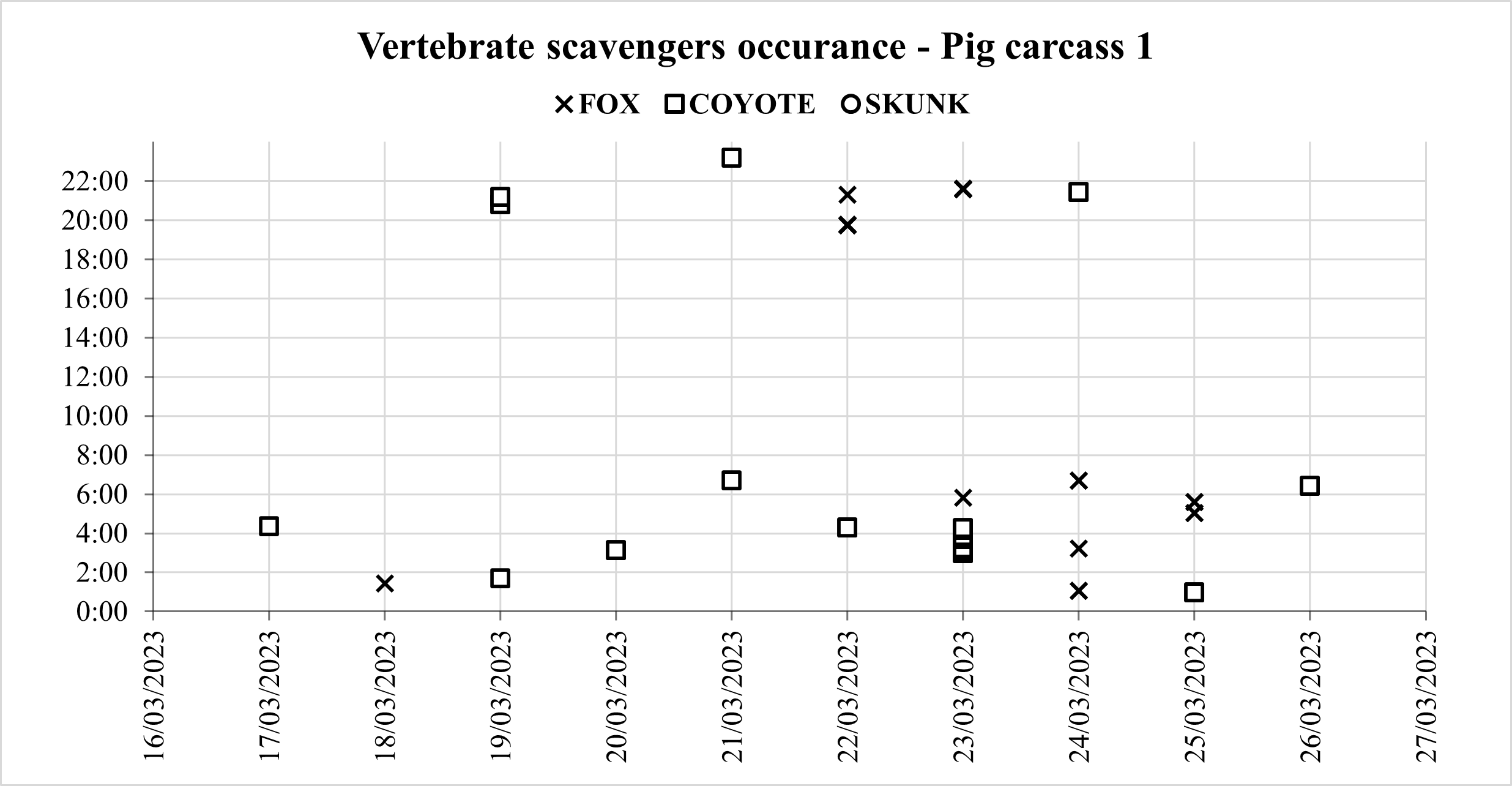

Supplement: Supplementary file 1 — Supplementary Material 1 [file 41598_2025_28834_MOESM1_ESM.tif]

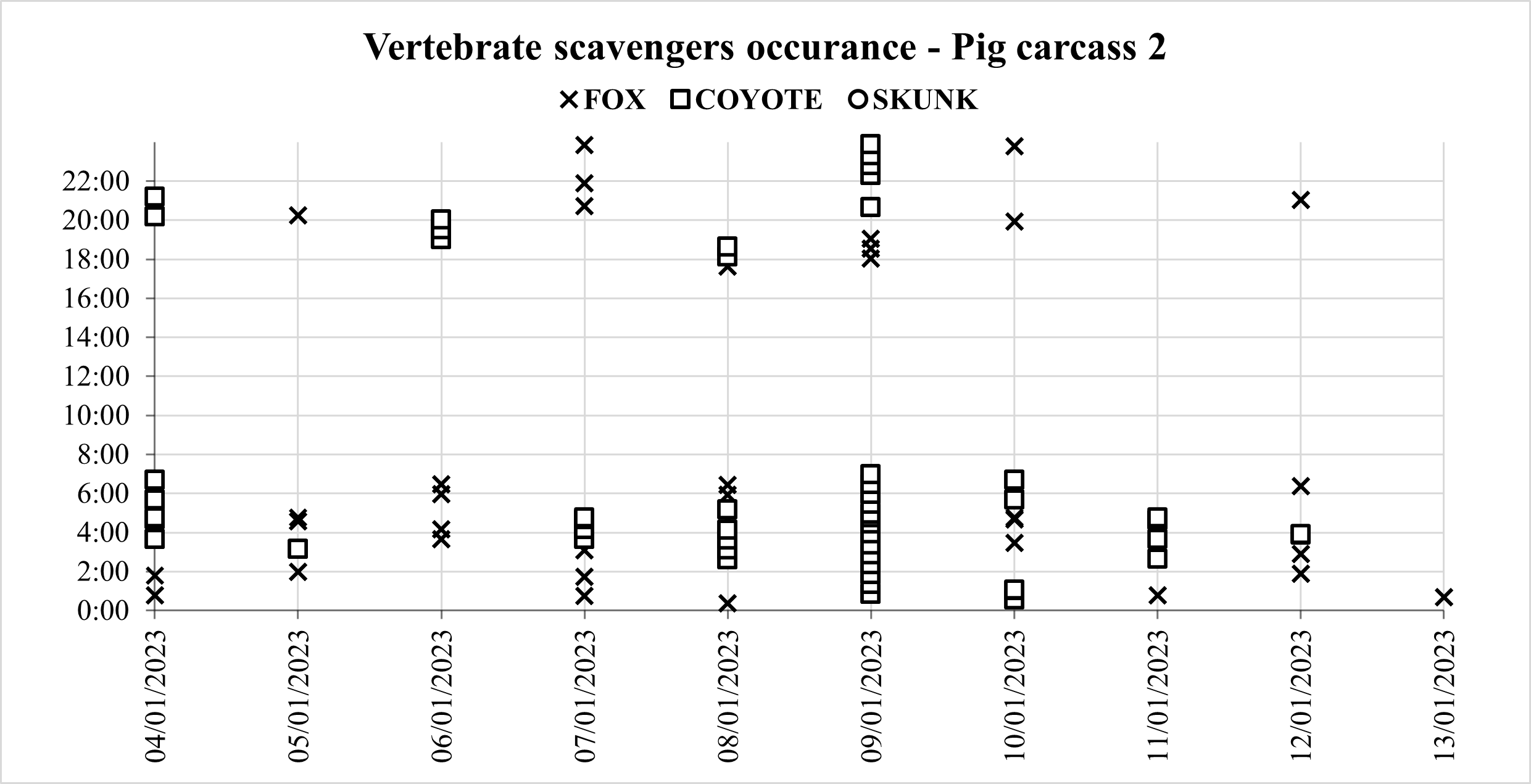

Supplement: Supplementary file 2 — Supplementary Material 2 [file 41598_2025_28834_MOESM2_ESM.tif]

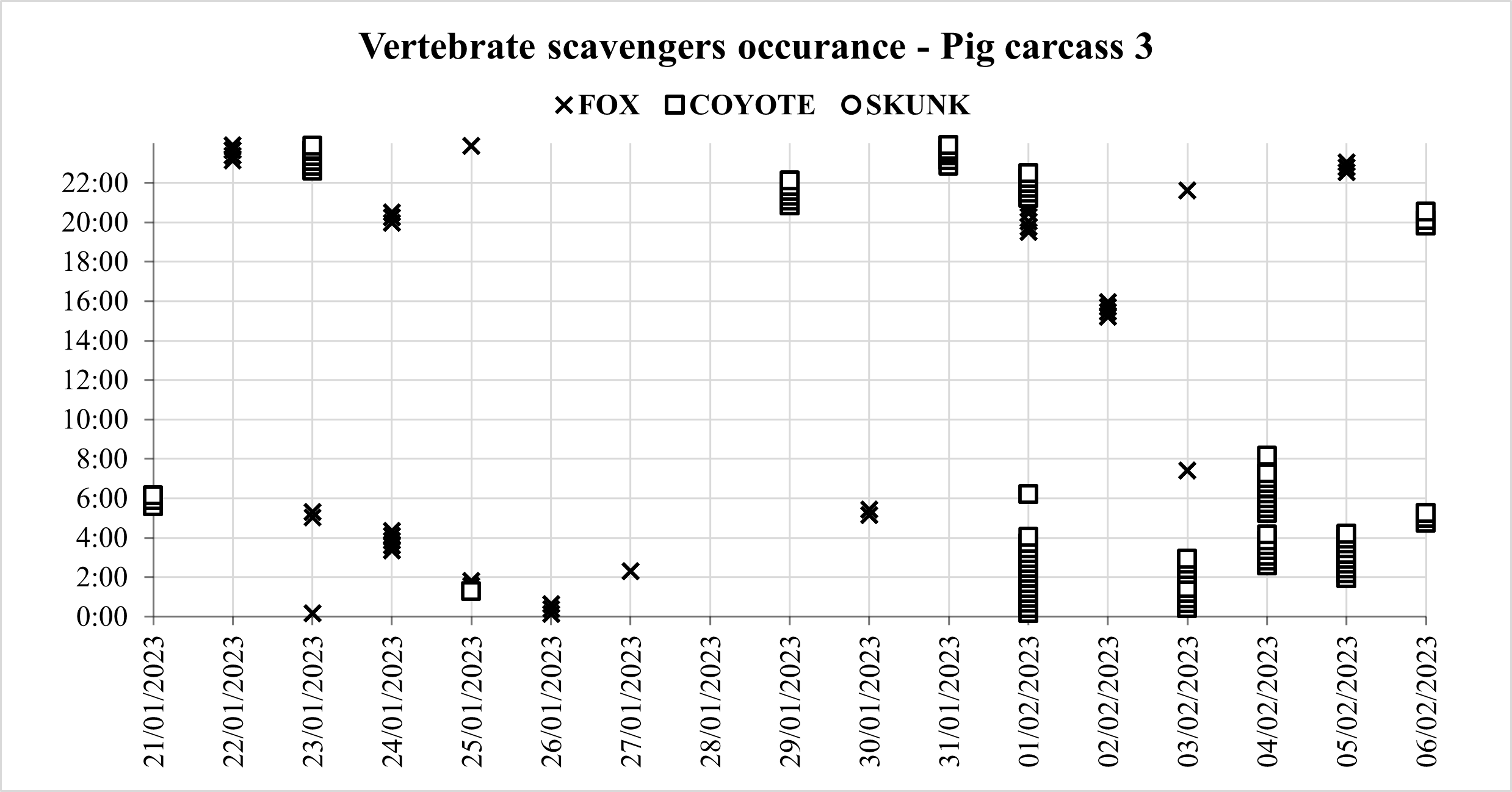

Supplement: Supplementary file 3 — Supplementary Material 3 [file 41598_2025_28834_MOESM3_ESM.tif]
